# ER-Bound Protein Tyrosine Phosphatase PTP1B Interacts with Src at the Plasma Membrane/Substrate Interface

**DOI:** 10.1371/journal.pone.0038948

**Published:** 2012-06-11

**Authors:** Melisa C. Monteleone, Ana E. González Wusener, Juan E. Burdisso, Cecilia Conde, Alfredo Cáceres, Carlos O. Arregui

**Affiliations:** 1 Instituto de Investigaciones Biotecnológicas, Universidad de San Martín, San Martín, Buenos Aires, Argentina; 2 Instituto de Investigación Médica Mercedes y Martín Ferreyra (INIMEC-CONICET), Córdoba, Argentina; University of Illinois at Chicago, United States of America

## Abstract

PTP1B is an endoplasmic reticulum (ER) anchored enzyme whose access to substrates is partly dependent on the ER distribution and dynamics. One of these substrates, the protein tyrosine kinase Src, has been found in the cytosol, endosomes, and plasma membrane. Here we analyzed where PTP1B and Src physically interact in intact cells, by bimolecular fluorescence complementation (BiFC) in combination with temporal and high resolution microscopy. We also determined the structural basis of this interaction. We found that BiFC signal is displayed as puncta scattered throughout the ER network, a feature that was enhanced when the substrate trapping mutant PTP1B-D181A was used. Time-lapse and co-localization analyses revealed that BiFC puncta did not correspond to vesicular carriers; instead they localized at the tip of dynamic ER tubules. BiFC puncta were retained in ventral membrane preparations after cell unroofing and were also detected within the evanescent field of total internal reflection fluorescent microscopy (TIRFM) associated to the ventral membranes of whole cells. Furthermore, BiFC puncta often colocalized with dark spots seen by surface reflection interference contrast (SRIC). Removal of Src myristoylation and polybasic motifs abolished BiFC. In addition, PTP1B active site and negative regulatory tyrosine 529 on Src were primary determinants of BiFC occurrence, although the SH3 binding motif on PTP1B also played a role. Our results suggest that ER-bound PTP1B dynamically interacts with the negative regulatory site at the C-terminus of Src at random puncta in the plasma membrane/substrate interface, likely leading to Src activation and recruitment to adhesion complexes. We postulate that this functional ER/plasma membrane crosstalk could apply to a wide array of protein partners, opening an exciting field of research.

## Introduction

PTP1B is a non receptor protein tyrosine phosphatase which associates to the cytosolic face of the endoplasmic reticulum (ER) through a hydrophobic tail of the C-terminal region [1]. The ER extensive network, and the topological orientation of PTP1B catalytic domain facing the cytosol, directs its activity to a variety of substrates [2]–[7]. Recent evidence also suggests that ER association with microtubules may contribute to PTP1B function in contact adhesion sites [8]–[10]. In most cases substrates of PTP1B were identified using substrate trapping mutants that stabilize the enzyme-substrate complexes, such as PTP1B-D181A [11]. Direct interaction between ER-bound PTP1B and endocytosed epidermal growth factor and platelet-derived growth factor receptors has been detected by Fluorescence Lifetime Imaging Microscopy [12], and by cryo-immuno electron microscopy [13]. In addition, Fluorescence Resonance Energy Transfer (FRET) and Bioluminiscence Resonance Energy Transfer (BRET) studies have shown that interactions between PTP1B-D181A and the insulin receptor (IR) occur in an endosomal compartment and during biosynthesis of the IR precursor at the ER [14], [15]. In contrast, interactions of PTP1B-D181A with targets at integrin and cadherin adhesion complexes, as well as with EphA3/ephrin-mediated cell-cell contacts seem to occur at the cell surface [8], [16]–[19].

An increasing body of research highlights PTP1B as a positive regulator of the non receptor protein tyrosine kinase Src in different cell models [8], [9], [16], [20]–[27]. Src is expressed in most cell types and its activity and subcellular distribution is tightly regulated [28], [29]. A myristoylation motif at the N-terminus mediates targeting of Src to the plasma membrane [30], and SH2 and SH3 motifs participate in its recruitment to cell-matrix adhesion sites [31]. However, Src localization and activity depend on its conformational state. In the inactive conformation, Src SH2 and SH3 domains engage in low-affinity intramolecular interactions, the SH2 domain with a phosphorylated tyrosine residue at the C-terminal region (Y529 in mouse Src), and the SH3 domain with a linker region between the SH2 and the catalytic domain of the kinase [32], [33]. Competition for the SH2 and SH3 domains and dephosphorylation of the C-terminal tyrosine and phosphorylation of a tyrosine in the activation loop, all contribute to Src activation [34].

Assessing the subcellular location where PTP1B dephosphorylates Src is important to understand Src activity regulation in intact cells. Previous work from our laboratory showed that PTP1B-D181A colocalized with Src family members at peripheral puncta, most likely associated with cell-matrix adhesion sites [8]. In addition, both PTP1B-D181A and wild type PTP1B co-immunoprecipitate with Src family kinases [8], [23], [24]. Although these studies suggest that PTP1B and Src may be engaged in common protein complexes they do not provide compelling evidence of direct physical interaction at the membrane-substrate interface. Bimolecular Fluorescence Complementation (BiFC) has emerged as a useful tool to study protein-protein interactions in cells [35]. Using this technique, a recent work in HEK293 cells explored multiple interactions of PTP1B with several target proteins, including Src [36]. This study, however, did not resolve where Src/PTP1B interactions occur in the cell and what are the structural interfaces implicated in the interaction.

In the present work, we analyzed the interaction between PTP1B and Src by BiFC at high spatial and temporal resolution. Fixed and living cells reveal BiFC puncta at the tip of ER tubules. Analysis of ventral membrane preparations by wide-field fluorescence as well as analysis of whole cells by total internal reflection fluorescence microscopy and surface reflection interference contrast reveal that BiFC puncta localize at the plasma membrane in contact with the substrate. These puncta are essentially abolished by replacing the regulatory C-terminal tyrosine residue (Tyr-529) of Src by phenylalanine. In addition, mutations disrupting the SH3 binding motif in wild type PTP1B reveal a secondary site of interaction. Our results show for the first time physical interactions of ER-bound PTP1B with Src at puncta localized at the plasma membrane in contact with the substrate, and further reveal that this interaction critically depends on the active site of PTP1B and the regulatory tyrosine 529 of Src. The results of the present paper illustrate a case of functional modulation in *trans*, among molecules located at the surface of the ER and plasma membrane, a phenomenon which may apply to a wide range of molecules and have impact in the regulation of several cell processes.

## Results

### Expression and Localization of BiFC Constructs

Several groups, including ours, have demonstrated that PTP1B dephosphorylates and activates Src in several cell models [9], [16], [20]–[27]. However, none of these biochemical studies address where this dephosphorylation and activation occurs in the cell. We sought to address this issue using the BiFC technique, which is based in the property of two non-fluorescent fragments of a fluorescent protein (YFP in this case, YN: amino acids 1–154, YC: amino acids 155–238) to form a fluorescent complex when they are brought together by the interaction of proteins fused to each fragment. BiFC allows visualization of protein complexes within living cells at high spatial resolution, without interference of fluorescence signals from non-bound proteins [35]. The limited temporal resolution of the technique, however, precludes its use for real-time detection of complex formation [37].

**Figure 1:**
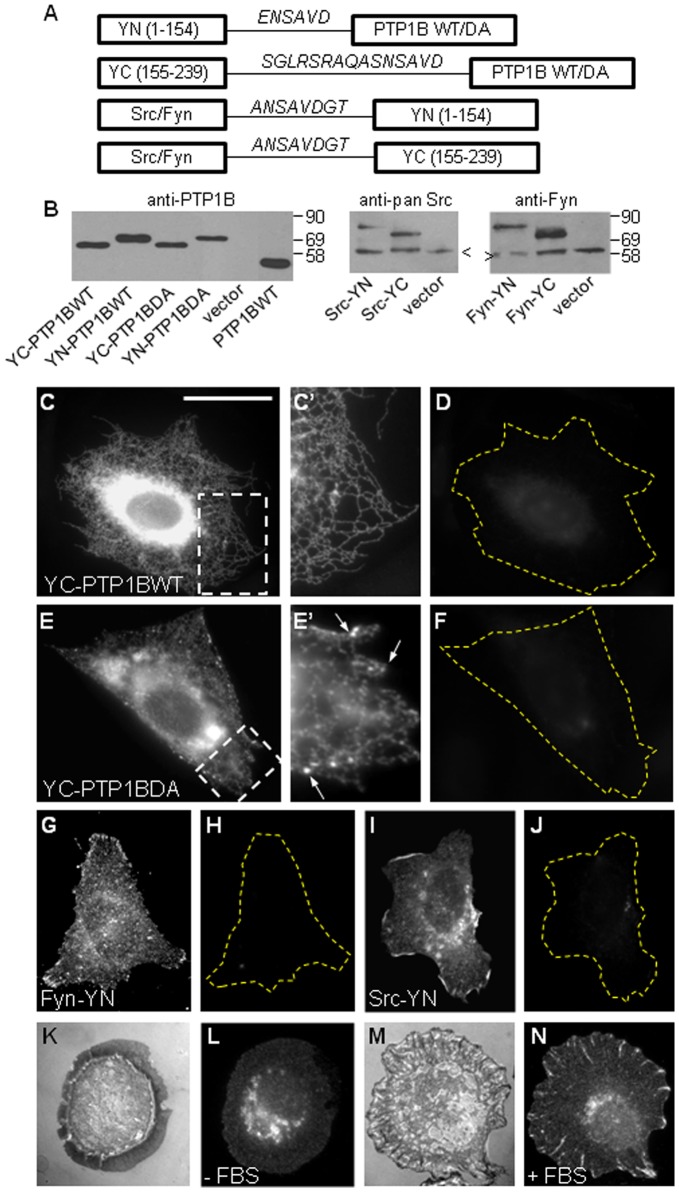
Expression and distribution of BiFC constructs. (A) Diagram of fusion proteins. YN (residues 1–154) and YC (residues 155–238) fragments of enhanced YFP were fused to the N-terminus of wild type (WT) and substrate trap mutant D181A (DA) PTP1B. The same fragments were fused at the C-terminus of mouse Src and Fyn. The amino acids of the linker region are indicated in italics. (B) Constructs were transiently expressed in CHO-K1 cells and probed in Western blots with anti-PTP1B (left panel), anti-Src (middle panel), and anti-Fyn (right panel) antibodies. YC and YN fragments of YFP add ∼10 and 18 kDa, respectively, to the partner fused proteins (PTP1B, Src and Fyn). Arrowheads indicate the migration of the endogenous proteins. Anti-PTP1B does not recognize the endogenous CHO-K1 protein; thus, a cell extract from PTP1B knockout cells reconstituted with human PTP1B was probed with this antibody and shown in the lane marked as PTP1BWT. Subcellular distribution of constructs used for BiFC was assessed by fluorescence microscopy. CHO-K1 cells expressing YC-PTP1BWT (C, C′, D), YC-PTP1BDA (E, E′, F), and SYF cells expressing Fyn-YN (G, H), and Src-YN (I, J) were immunolabeled with anti-PTP1B (C, Ć, E, É), anti-Fyn (G) and anti-Src (I) followed by secondary antibodies conjugated with Alexa Fluor 568 nm. Images on the red channel show that YC-PTP1BWT (C, Ć) and YC-PTP1BDA (E, É) localize in the ER, as expected (Ćand É are magnifications of regions within boxes in C and E, respectively). In addition, YCPTP1BDA accumulates in small puncta (arrows in É). Fyn-YN (G) and Src-YN (I) are enriched at the cell margin and in a perinuclear compartment. All constructs display background fluorescence at the green channel in which BiFC is analyzed (D, F, H, J). (K-N) Starved SYF cells expressing Src-YN were plated for 30 min in the absence (K, L) and in the presence (M, N) of serum. Note that Src-YN localizes in a perinuclear compartment in the absence of serum (L) and redistributes to peripheral, radial focal adhesions in the presence of serum (N), as expected. (K, M) Surface reflectance interference contrast images showing the membrane in contact with the substrate. Dashed lines indicate the perimeter of cells. Scale bar, 40 µm.

Different combinations of fusion proteins were generated for BiFC analysis (Figure 1A). YN (residues 1–154) and YC (residues 155–238) fragments of enhanced YFP were added to the N-terminus of PTP1B, which did not significantly affect its targeting to the ER [8], [16], [36]. We prepared fusions to both, the wild type (WT) enzyme and the substrate trapping mutant PTP1B-D181A (DA) [11]. PTP1BDA increases significantly the steady state population of PTP-substrate complexes, feature that has been proved to be crucial for detection of PTP1B and growth factor receptors interactions by using optical techniques [12], [14], [15]. In the case of Src and Fyn kinases, YC and YN fragments were added to their C-terminus, which has been proved not to affect their membrane targeting and activation [38], [39]. To evaluate the expression of fusion proteins all constructs were transiently transfected in CHO-K1 cells and analyzed by Western blots using specific antibodies. All fusion proteins expressed and migrated at the expected molecular weights (Figure 1B). Their distribution in cells was evaluated by immunofluorescence, and as expected, YN fusion (not shown) and YC-PTP1BWT, showed typical ER distributions (Figure 1, C and C′). The YC-PTP1BDA construct localized in the ER, and in specific regions of this compartment accumulated and formed bright puncta (Figure 1, E and E′). These PTP1B distributions resembled those seen using the full-length GFP as a fusion protein [8], [12], [16].

The distribution of Src and Fyn fusions was assessed, by immunofluorescence, in CHO-K1 and in SYF cells, with essentially the same results. Here we show the results in SYF cells, which lack the endogenous expression of both kinases. YC (not shown) and YN fusions displayed similar distribution patterns. Fyn-YN showed a grainy fluorescence distributed all over the cell but it was more intense at the cell border, consistent with a plasma membrane distribution (Figure 1G) [40], [41]. Src-YN showed a strong immunolabeling at the cell border and at the perinuclear compartment, consistent with a plasma membrane and endosomal localization (Figure 1I) [38], [42]–[45]. The distribution of Src was shown to correlate with its activation state, which in turn can be modulated by cell stimulation [43], [44]. Under starvation conditions, most of Src is inactive and localizes at the perinuclear compartment. In contrast, the open conformation of active Src accumulates in focal adhesions at the cell periphery. In agreement with this, immunofluorescence analysis of Src-YN expressed in serum-starved SYF cells showed a strong signal at the perinuclear region (Figure 1L). Acute stimulation with serum, revealed a partial re-distribution of the signal to focal adhesions at the cell periphery (Figure 1N). In all cases, the immunolabel was detected in the red channel. The intrinsic fluorescence of YN and YC fragments was assessed in the yellow/green channels and always showed background levels (Figure 1, D, F, H and J).

### BiFC Reveals Interactions between PTP1B and c-Src in Puncta Associated to the ER

**Figure 2:**
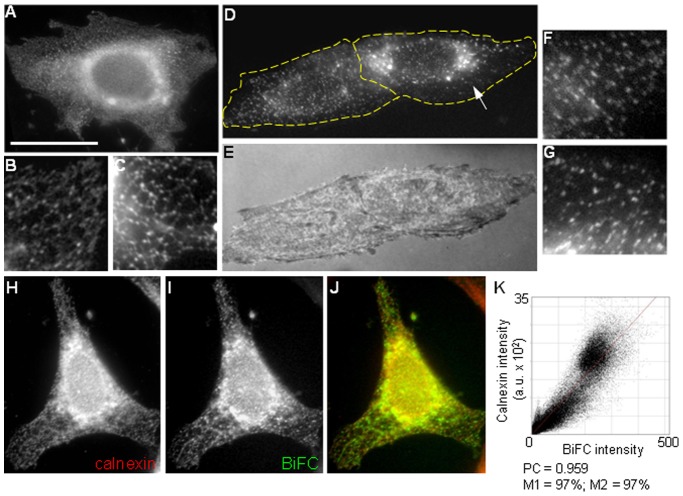
Distribution of the BiFC signal. CHO-K1 cells were co-transfected with BiFC pairs and analyzed by fluorescence microscopy. (A-C) Representative BiFC distribution of YC-PTP1BWT/Src-YN is shown. Most cells show the BiFC signal as bright fluorescence puncta associated with a network pattern of lower fluorescence intensity (A, magnifications in B and C). (D–G) Representative BiFC distribution of YC-PTP1BDA/Src-YN is shown. Note that BiFC is exclusively seen as bright puncta (magnifications in F, G), sometimes more dense in the perinuclear region (arrow in D). Scale bar in A: 25 µm. Magnifications in B, C, F, and G are at 200% of the original images (E) Image taken under surface reflection interference contrast. (H–J) Representative CHO-K1 cell co-transfected with the YC-PTP1BWT/Src-YN pair and then fixed and processed for immunofluorescence detection of calnexin, using Alexa Fluor 568-conjugated secondary antibodies. (H) Calnexin labeling, (I) BiFC, (J) merge of both channels. (K) Cytofluorogram showing the high correlation between red/green pixels corresponding to the calnexin and BiFC images, respectively. Arbitrary units (a.u.) represent grey level values from 12-bit images. Pearson’s correlation coefficient close to 1 reveals positive correlation. Manders’ coefficients M1 and M2 estimate the amount of co-localizing signal from the calnexin image to the BiFC image and viceversa, respectively. Both M1 and M2 coefficients are close to 100% indicating an almost perfect co-localization. Dashed lines indicate the perimeter of cells.

From all BiFC pairs tested, only Src-YN in combination with YC-PTP1BWT and YC-PTP1BDA produced a detectable fluorescence over background levels. For Src-YN/YC-PTP1BWT pair, BiFC distributed, in most of the analyzed cells (86%), as bright fluorescent puncta embedded in a reticular pattern of weaker fluorescent intensity (Figure 2, A-C and Table 1). BiFC distribution tightly overlapped with that of calnexin, confirming its ER localization (Figure 2, H-J). Quantitative estimation of co-localization of BiFC and calnexin was further performed using the JACoP plugin of ImageJ [64] (See Materials and Methods). The distributions of red (calnexin)/green (BiFC) pixels in a scatter plot revealed a positive correlation between both signals (Figure 2K). Pearson correlation (PC) coefficient in more than 20 cells was >0.9, being 1 in case of a perfect co-localization [64]. Manders coefficients M1 and M2 also were >0.9 which means that more than 90% of both signals co-localized. Less frequently (14% of cells), BiFC signal was seen exclusively as puncta (Table 1). When the substrate trap mutant D181A (DA) was used instead of wild type PTP1B, BiFC signal was exclusively seen as bright puncta (Figure 2, D-G). These puncta overlapped with calnexin (not shown) and uniformly spread throughout the entire area of the cell, although occasionally appeared more concentrated at the perinuclear region (Figure 2D, arrow). The size of BiFC puncta measured at thin lamellar regions ranged from 0.4–0.6 µm in diameter. In some experiments, either Src or PTP1B were labeled with antibodies to examine the relationship among the intensity of the BiFC signal and Src/PTP1B expression levels. BiFC signal occurred at expression levels of Src and PTP1B that are close to the endogenous levels (Figure S1, only Src label is shown). In addition, similar BiFC signal was achieved in cells expressing different levels of the BiFC pairs. These results indicate that BiFC signal saturates at low levels of Src and PTP1B expression.

**Table 1:** Expression of the BiFC signal in wild type and mutant forms of Src and PTP1B.

	ER + punctate (%)	Only punctate (%)	n
PTP1BWT/SrcWT	86	14	257
PTP1BPA/SrcWT	43	18	219
PTP1BWT/SrcYF	13	0	147
PTP1BPA/SrcYF	14	4	141
PTP1BDA/SrcWT	1	99	147
PTP1BDAPA/SrcWT	0	94	132
PTP1BDA/SrcYF	0	34	265
PTP1BDAPA/SrcYF	0	10	195

BiFC signal was assessed in cells that were positive for the detection, by immunofluorescence, of a member of the BiFC pair. Three categories were observed in the BiFC channel: background (not shown), ER + punctate, and only punctate. n: number of analyzed cells.

Fyn, another ubiquitous member of the Src family, regulates a variety of signal transduction processes in common with Src [46]. To determine if PTP1B was able to interact with Fyn, we prepared a Fyn-YN construct and analyzed BiFC production and distribution. CHO-K1 cells expressing both pairs, YC-PTP1BWT/Fyn-YN and YC-PTP1BDA/Fyn-YN, showed a positive BiFC signal, which distributed in puncta throughout the cell (Figure S2).

We were intrigued with the compartments involved in BiFC puncta formation and we sought to characterize them further. It was previously shown that a subpopulation of Src colocalizes with rab11-containing endosomes in the perinuclear region [38]. To determine if BiFC puncta at the perinuclear region co-localize with a rab11 recycling compartment, we co-transfected CHO-K1 cells with the YC-PTP1BDA/Src-YN BiFC pair and mRFP-rab11. As expected, the signal of mRFP-rab11 was displayed as puncta dispersed throughout the cell, with higher intensity around the perinuclear region. Inspection for co-localization of the fluorescent images at the microscope and quantitative analysis using JACoP did not reveal any significant overlap between BiFC puncta and mRFP-rab11 (Figure S3, A–D).

If BiFC puncta were associated to traffic carriers they would be expected to display directional movements along the cytoplasm. To examine this issue we determined the position of BiFC puncta in series of time-lapse analysis. In live cells, BiFC using Src-YN with YC-PTP1BWT or YC-PTP1BDA revealed distribution patterns essentially identical to those described for fixed cells (Figure S3, E, F). Since BiFC puncta using YC-PTP1BDA displayed a higher signal/noise ratio than those observed using YC-PTP1BWT, YC-PTP1BDA was considered to be more suitable for time lapse imaging, particularly because the preservation of the signal requires important attenuation of the excitation light and short exposure times. We could not detect a significant degradation of the signal in our imaging conditions (Figure S3, I, and plots a, a′). Time-lapse data revealed that most BiFC puncta exhibited random movements within a limited area (<1 µm^2^) and did not move in any specific direction (Figure S3, J, movie S1). During the 10 minutes period of the analysis it was evident that a fraction of fluorescent puncta appeared and disappeared from the field. Quantitative analysis at higher magnification revealed that emergence of new BiFC puncta was frequently accompanied by reduction of the fluorescence intensity of adjacent ones, suggesting that puncta emerge as consequence of splitting events (Figure S3, J, plot b, b′). In addition, analysis of high magnification images in function of time revealed that BiFC puncta were at the tips of dynamic tubules, which seem to emerge from the ER network (Figure S3, J, movie S1). In fact, time-lapse analysis of GFP-PTP1BDA revealed similar formation of dynamic puncta connected to the network of ER tubules (Figure S4, C). We also sought to determine whether BiFC puncta co-localized with sec24, a marker of ER exit sites, which also displays a punctate distribution on the ER similar to the pattern observed for BiFC puncta [48]. Qualitative and quantitative co-localization analysis performed in live cells did not show correlation between BiFC puncta and mRFP-sec24 (Figure S3, G and H). Similar results were obtained from analyses of time-lapse series (not shown).

### Punctate BiFC Signals Localize at the Membrane-substrate Interface

**Figure 3:**
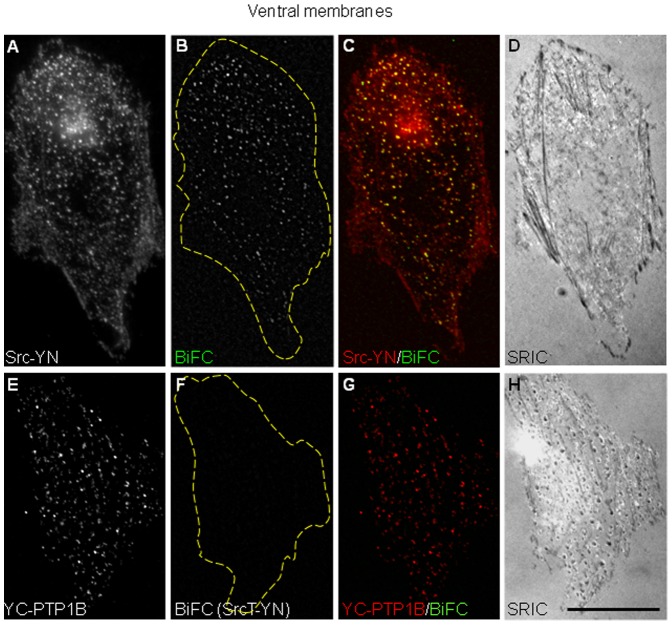
BiFC in ventral membranes. CHO-K1 cells were co-transfected with YC-PTP1B and either Src-YN (A–D) or SrcT-YN (E–H). Membrane preparations were obtained by sonication of the cells previously exposed to hypotonic conditions. After fixation with paraformaldehyde, Src-YN (A) and YC-PTP1B (E) were detected by specific primary antibodies and Alexa Fluor 568-conjugated secondary antibodies. The BiFC signal was displayed in puncta that tightly colocalized with the Src-YN staining (C). In contrast, BiFC signal was undetectable when using SrcT-YN (F, G). SRIC analysis showed dark/light patterns of the ventral membrane in contact with the substrate (D, H). Dashed lines indicate the perimeter of cells. Scale bar: 25 µm.

**Figure 4:**
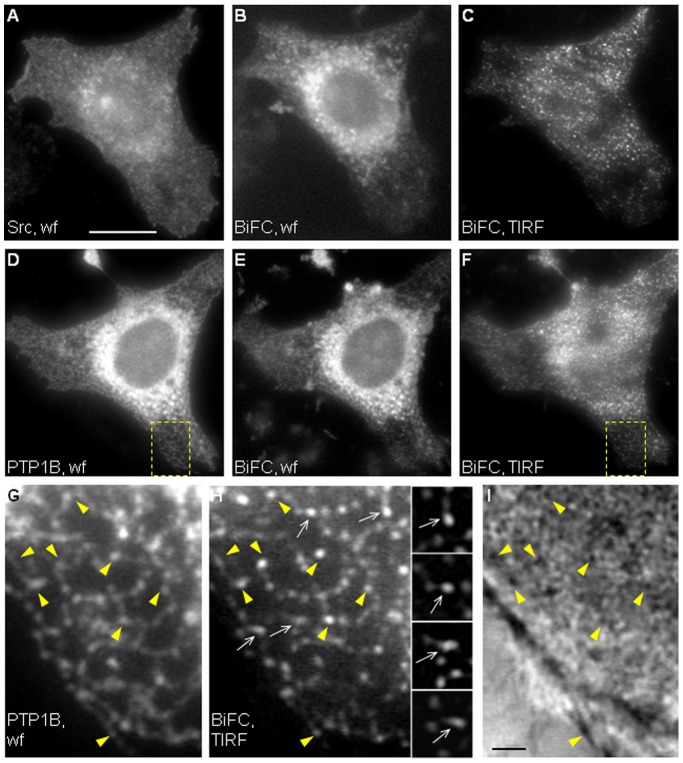
BiFC analysis by TIRFM. CHO-K1 cells were co-transfected with YC-PTP1BWT/Src-YN and then fixed and processed for immunofluorescence detection of Src (A) and PTP1B (D) by wide field (wf). The BiFC signal of the same cells was observed by wide field (B, E) and under TIRF illumination (C, F). Yellow boxes in D and F were magnified in G and H. Note that BiFC puncta visualized under TIRF illumination (H) overlap with the ER network seen with anti-PTP1B (G). Also note that some puncta (white arrows in H and insets) display a comet-like appearance with a grading intensity of fluorescence brightest at the tip, suggesting a “dipping down” toward the substrate and into the region of exponentially increasing excitation of the evanescent field. Several BiFC puncta (yellow arrowheads) coincide with dark spots visualized by SRIC (I). Scale bar in A, 25 µm; scale bar in I, 2.5 µm.

The absence of co-localization of BiFC puncta with rab11, their lack of directional movement, and their visible association with dynamic ER tubules, suggest that they could arise from point interactions between ER-bound PTP1B and a pool of Src localized at the plasma membrane. This view is consistent with the exclusive BiFC puncta pattern seen using Fyn, which is a bona fide plasma membrane Src family member (Figure S2). Src associates with the plasma membrane through a myristoylation domain at the N-terminus [29], [30], [45], [47]. To determine whether BiFC occurred at the membrane in contact with the substrate, we prepared ventral membranes from YC-PTP1BDA/Src-YN transfected cells. Ventral membranes were prepared using well-established protocols [62], [63]. As expected, the procedure removed nuclei and most of the cytoskeleton and ER, leaving behind the ventral membrane attached to the substrate (Figure S5, C–F). Using antibodies for Src and PTP1B detection, we found that expression of Src-YN and YC-PTP1BDA puncta were retained within ventral membranes (Figure S5, G and H). More important, Src-YN and YC-PTP1B also showed the presence of BiFC in ventral membranes (Figure 3, B and C). These experiments were repeated with SrcT-YN, which was truncated at the N-terminus, lacking 12 residues which included the myristoylation target site, the glycine 2, and the polybasic motif conformed by lysines 5, 7 and 9, both motifs essential for membrane association [30]. BiFC was greatly reduced in ventral plasma membranes when SrcT-TN was used (Figure 3F). This result indicates that BiFC depends critically on Src association to the ventral membrane. Indeed, we confirmed that SrcT was not retained in ventral membrane preparations while most of a membrane-targeted Lck-mCherry did (Figure S6). These results suggest that BiFC between Src-YN and YC-PTP1BDA or YC-PTP1B occurs in puncta at the plasma membrane in contact with the substrate, and are in agreement with previous findings of our laboratory which showed that ER-bound GFP-PTP1B is dynamically positioned at the cell periphery in fibroblasts and hippocampal neurons [8], [9]. To further determine if BiFC puncta occurred at the cell/substrate interface we analyzed cells by total internal reflection fluorescence microscopy (TIRFM) [49]. The incident light under TIRFM produces the excitation of fluorophores within an evanescent field of decreasing energy, maximum at the substrate interface, which in our system had a depth of ∼210 nm (see Materials and Methods). Examination of CHO-K1 cells expressing YC-PTP1BWT/Src-YN pair under TIRFM revealed BiFC puncta scattered all over the cell area (Figure 4, C and F). High magnification analysis revealed that these puncta overlapped with the ER network, visualized by PTP1B labeling under wide field epifluorescence (Figure 4, D, G, and H). BiFC puncta frequently displayed a comet-like appearance with a grading intensity of fluorescence which was brightest at the tip, suggesting a “dipping down” of puncta toward the substrate, into the region of exponentially increasing excitation of the evanescent field (Figure 4H, insets). Surface reflection interference contrast (SRIC) microscopy reveals the specimen’s proximity and adherence to the substrate within the nanometer range, and dark and light patterns represent membrane regions approaching and separating from the substrate, respectively [50]. We always found a fraction of BiFC puncta overlapping with dark spots seen by SRIC, suggesting that BiFC puncta at the ventral membrane may form in sites of close contact with the substrate (Figure 3I, arrowheads). To determine BiFC distribution in puncta and “comets” under TIRFM corresponds to that of ER-bound PTP1B, we analyzed the distribution of GFP-PTP1B by TIRFM. The results were essentially identical to those described for BiFC (Figure S4, A–B″).

### Structural Determinants Required for BiFC between PTP1B and c-Src

**Figure 5:**
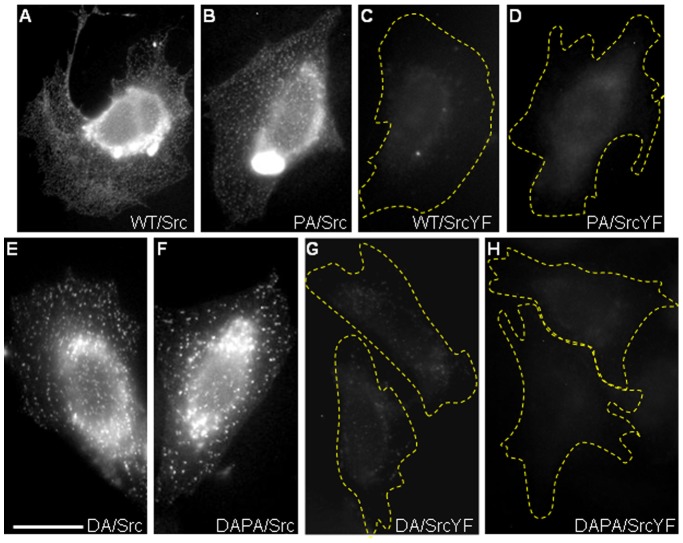
BiFC distribution using PTP1B and Src mutants. CHO-K1 cells were co-transfected with different BiFC pairs and then fixed and processed for detection of Src or PTP1B using Alexa Fluor 568-conjugated secondary antibodies. Only images of the green channel corresponding to the BiFC signal are shown. (A) YC-PTP1BWT/Src-YN, positive control of BiFC; (B) YC-PTP1BPA/Src-YN, PA designates a proline to alanine mutation of PTP1B (P309/310A) which disrupts the SH3-binding motif; (C) YC-PTPBWT/SrcYF-YN, YF designates a substitution of the tyrosine 529 of Src by phenylalanine; (D) YC-PTP1BPA/SrcYF, this tests the effect of combining both PA and YF mutations; (E) YC-PTP1BDA/Src, positive control of BiFC using the substrate trapping mutant D181A; (F) YC-PTP1BDAPA/Src-YN, this tests the disruption of the SH3-binding motif (PA mutation) in the context of the substrate trap PTP1B (DA mutation); (G) YC-PTP1BDA/SrcYF-YN; (H) YC-PTP1BDAPA/SrcYF-YN. Note that elimination of the tyrosine 529 at the C-terminal region of Src is the only mutation reducing significantly BiFC production. Dashed lines indicate the perimeter of cells. Scale bar, 20 µm.

To identify the motifs involved in the interaction between PTP1B and Src we introduced mutations in specific residues of PTP1B and Src. Substitution of prolines 309 and 310 to alanines (PA) in the SH3 type II binding motif present in PTP1B effectively disrupts PTP1B interaction with the SH3 domain of Src in *in vitro* binding assays [22]. Targeting of YC-PTP1B to the ER was not affected by introducing the P309/310A mutations (not shown) [8]. The BiFC signal produced by YC-PTP1BPA/Src-YN pair followed a punctate distribution pattern similar to that produced by PTP1B WT, although there was an overall decrease of the signal intensity, which reached background levels in 39% of the cells (Figure 5B, Table 1).

PTP1B modulates Src activity by dephosphorylation of a regulatory tyrosine at the C-terminus of Src (tyrosine 529 in mouse Src), removing the negative constraint imposed by the intramolecular interaction between the phosphorylated state of this residue and the SH2 domain [16], [20]–[25]. PTP1B binding to this tyrosine might be necessary to stabilize the interaction required for BiFC, mainly in the context of PTP1B substrate trap. To evaluate this possibility we replaced Src tyrosine 529 by phenylalanine. The cellular localization of SrcY529F-YN construct, assessed by Src immuno-labeling, had the expected distribution, with noticeable accumulation at peripheral focal adhesions (not shown) [38], [43]. In cells expressing the YC-PTP1BWT/SrcY529F-YN pair the BiFC signal was significantly reduced to background levels (Figure 5C, Table 1). A similar result was obtained in cells expressing both mutations YC-PTP1BPA/SrcY529F (Figure 5D, Table 1). Introducing PA mutations into the substrate trapping DA enzyme, PTP1BDAPA, did not have a noticeable effect on BiFC signal (Figure 5F, Table 1). However, the combination of either YC-PTP1BDA or YC-PTP1BDAPA with Src mutant SrcY529F-YN reduced BiFC signal to background levels (Figure 5, G and H, Table 1).

## Discussion

In the present paper we used the BiFC technique to demonstrate direct physical interactions among ER-bound PTP1B and tyrosine kinases Src and Fyn at the plasma membrane. These interactions were revealed as bright fluorescence puncta associated with the ER. Our results strongly suggest that PTP1B, which is localized at the tip of dynamic ER tubules, was positioned close to the ventral membrane in contact with the substrate and interacted with Src at multiple puncta sites. We further show that mutations altering the active site of PTP1B and removing the negative regulatory residue of Src (by replacing tyrosine 529 by phenylalanine) significantly reduced BiFC complex formation. These results suggest that ER-bound PTP1B releases Src from its negative regulation at random point contacts of the membrane/substrate interface, leading to its activation and possibly recruitment to adhesion complexes [8].

### Subcellular Distribution of BiFC

The subcellular localization of BiFC puncta is expected to be conditioned by the subcellular localization of each interaction partner. PTP1B is anchored to the cytosolic face of the ER membrane through a hydrophobic C-tail, allowing its mobility through the vast surface of the ER, and its interaction with substrates in the cytosol. In addition, dynamic changes of shape of the ER, dependent on its association with microtubules [9], [51]–[53], extend the range of PTP1B interactions to a larger spatial scale, positioning PTP1B in the cell cortex, and therefore facilitating its encounter with substrates associated with the cytosolic face of the plasma membrane. Among these potential substrates, several laboratories including ours have identified the Src family of tyrosine kinases [9], [16], [20]–[27]. Src kinases associate with plasma membrane by means of fatty acid modifications, and by protein-protein interactions [30], [43], [47]. In addition, a fraction of Src remains in the cytosol and another is associated with recycling endosomes [29], [42], [45], [47]. On this regard, the weak BiFC signal revealing the ER network, when using the wild type PTP1B, likely represents interactions with the freely diffusing pool of cytosolic Src. In contrast, bright BiFC puncta in the ER likely reflects interactions with spatially restricted Src and PTP1B molecules. We found that BiFC puncta did not co-localize with rab11, a recycling endosomes marker, and did not display directional movement in the cytoplasm, as expected for traffic carriers (Figure S3). Therefore, it is unlikely that BiFC puncta reflect interactions with an endosomal pool of Src. The fact that BiFC puncta were retained in ventral membrane preparations, were visualized within the evanescent field produced by TIRF microscopy, and some co-localized with dark spots seen under SRIC microscopy, strongly suggest that they could represent spatially restricted interactions of ER-bound PTP1B with a subset of Src localized at the plasma membrane. Indeed, BiFC was significantly reduced when SrcT-YN was used. A recent FRAP beam-size analysis revealed that membrane-bound wild type Src-GFP has lateral diffusion rates similar to lipid probes, a property impaired in active Src-Y527F-GFP mutant, presumably due to interactions with other membrane proteins in a Src SH2-dependent manner [47]. Interestingly, we found that BiFC puncta were significantly reduced when the Y529F mutant of Src (the equivalent to Y527F in chicken Src) was analyzed (Figure 5, Table 1), suggesting that PTP1B targets plasma membrane Src on this residue (see further discussion below).

**Figure 6:**
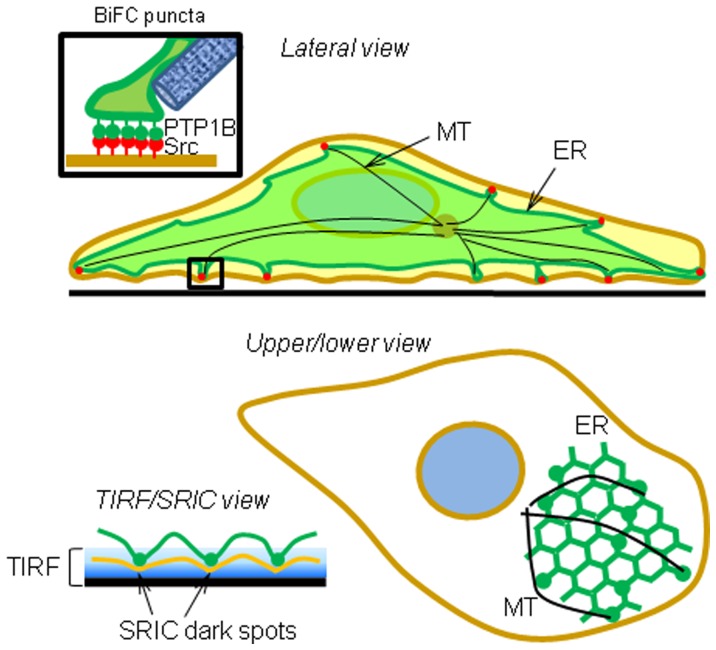
Schematic view of BiFC results. ER membranes (green) are positioned close to the plasma membrane (brown) by microtubules (black). PTP1B anchored to the cytosolic surface of the ER membrane (green pins) can interact with Src (red pins) associated to the cytosolic side of the plasma membrane. The substrate trap mutant PTP1BDA enhances the interaction leading to the visualization of large puncta (inset, and green beads on the ER in the upper/lower view). TIRF microscopy reveals BiFC puncta at the ventral membrane and SRIC shows that some of BiFC puncta occur in close contact with the substrate (SRIC dark spots).

Our time-lapse experiments showed that BiFC puncta move apparently at random and locate at the tip of ER tubules (Figure S3), and under TIRFM they were frequently visualized as bright spots at one tip of comet-like fluorescent structures (Figure 4), suggesting a “dipping down” of ER tubules towards the plasma membrane in contact with the substrate. Parallel analysis of GFP-PTP1BDA showed similar results (Figure S4) suggesting that PTP1B localization at the tip of ER tubules imposes a restriction to the spatial propagation of the interaction with Src molecules associated to the cytosolic side of the plasma membrane. A schematic view derived from our results is shown in Figure 6. ER-bound PTP1B positioning in the cell cortex requires microtubules [8], [9] (González Wusener et al., unpublished results). The comet-like fluorescent figures observed under TIRFM suggest that ER tubules approach to the membrane at different angles, as previously shown for microtubules [54]. In the context of the substrate trapping mutant PTP1BDA, interactions with plasma membrane-associated Src are stabilized, and therefore BiFC puncta enhanced (represented by green spheres in Figure 6). Mechanisms underlying the fusion and split of BiFC puncta (Figure S3), which were also seen for GFP-PTP1B (Figure S4), are currently unknown, but likely depend on the dynamics of ER tubules [55], [56].

Our analysis of BiFC was extended to YC-PTP1BWT/Fyn-YN and YC-PTP1BDA/Fyn-YN pairs. In both cases we found a positive BiFC signal that distributes in puncta, as it was described for Src. Fyn has a more tight association to the plasma membrane than Src due to additional palmitoylation (which does not occur in Src), and quickly associates with the plasma membrane after being synthesized [57]. This strengthens the view that BiFC puncta between PTP1B and Src kinases most frequently occur in association with the plasma membrane. However, we cannot completely rule out transient interactions between PTP1B and Src within the endosomal compartment. To elucidate this, a more extensive co-localization analysis with markers for different endosomes would be required, as well as high resolution double time-lapse analyses.

### Molecular Determinants of BiFC Puncta in PTP1B and Src

In our study we showed that a truncation of the Src N-terminus, which removes the myristoylation target site and the polybasic motif involved in membrane association, eliminates the production of BiFC. Thus, membrane-bound Src is a requisite for BiFC to occur. Myristate is added to Src cotranslationally by the N-myristoyl-CoA-protein transferase enzyme [30]. Beyond that, little is known about the regulation of Src myristoylation. We identified the active site of PTP1B and the Src tyrosine 529 at the C-tail as major determinants underlying BiFC (Figure 5, Table 1). Tyrosine 529 is phosphorylated by the C-terminus Src kinase, Csk [58], and dephosphorylated by PTP1B and other tyrosine phosphatases [16], [20]–[26], [65]. Replacement of tyrosine 529 by phenylalanine significantly reduced but did not completely eliminate BiFC puncta (Table 1). In addition, a single mutation (D181A) converting the PTP1B active site in a substrate trap significantly enhanced BiFC puncta throughout the cell, provided that the tyrosine 529 of Src remained unchanged. A second determinant contributing to BiFC is a proline-rich motif of PTP1B which fits the consensus sequence for class II SH3 domain-binding motifs [59]. Using PTP1BPA, a proline mutant in which the SH3-binding motif was disrupted [8], [59], moderately reduced the BiFC signal (Figure 5, Table 1). Remarkably, PA mutation had no effect when combined with the substrate trap mutation DA (PTP1BDAPA). These results suggest that PTP1B SH3-binding motif contributes to stabilize weak interactions that are enough for BiFC to occur. However, the presence of a substrate trap mutation (i.e. DA) which stabilizes the active site binding to the substrate turns the SH3-binding motif contribution essentially irrelevant. This view is compatible with binding affinity determinations, which for SH3 domains to their peptide ligands (∼10 µM) is significantly lower than that of PTP1BDA to their phosphorylated peptides (∼0.04 µM) [60], [61].

On the other hand, in previous reports we have shown that impairing microtubule dynamics and distribution abolished PTP1B positioning to the cell periphery [8],[9]. Thus, we predict that BiFC would not be produced if microtubule distribution and dynamics are affected by artificial means.

In conclusion our work provides new and detailed molecular information revealing that ER-bound PTP1B is capable of interacting with Src kinase at point contacts established between the ER and the plasma membrane in the cell/substrate interface. Our data suggest that Src association to the plasma membrane, through the N-terminus myristoylation and polybasic motifs, is essential for BiFC to occur. In the membrane, PTP1B targets tyrosine 529 at the Src C-tail, unlocking the negative regulation imposed by the phosphorylation of this residue.

## Materials and Methods

### DNA Constructs

DNA sequences coding for the N-terminal (YN) and C-terminal (YC) regions of YFP were amplified by PCR from pEYFP-C1 plasmid (Clontech BD Biosciences, Mountain View, CA). YN contained amino acids 1–154 and YC amino acids 155–238. To add YN/YC at the N-terminus of human PTP1B, the Age I/Eco RI YN fragment and the Age I/Sal I YC fragment were introduced in frame and replaced full length EYFP in pEYFP-C1-PTP1B WT or D181A (DA) giving YN/YC-PTP1B WT/DA (see Figure 1A; [8]). Mouse Src cDNA was provided by S. Shattil (University of California at San Diego) and Fyn cDNA was provided by Jon Cooper (Fred Hutchinson Center, Seattle). They were introduced into the Xho I/Eco RI sites of phCMV3 (Gene Therapy Systems, Inc. San Diego, USA). To add YN and YC at the C-terminus of Src and Fyn kinases, Kpn I and Bam HI sites were introduced to the 5′ end of sense and antisense primers, respectively, and the PCR fragments were cloned into the same sites of phCMV3-Src/Fyn to obtain Src/Fyn-YN/YC. Truncation of the twelve N-terminus amino acids of Src was carried out by PCR, using as a template Src-YN. Xho I and Eco RI sites were added at the 5′ end of the truncation sense primer and the antisense primer, respectively. The PCR product, SrcT-YN, was inserted into Xho I/Eco RI of phCMV3. All constructs were verified by sequencing. Proline mutants (prolines 309 and 310 replaced by alanines) of PTP1B were obtained by PCR, as described before [8]. PTP1B PA and DAPA mutants in pEGFP-C1 vector were excised using Sal I/Xba I restriction enzymes and replaced PTP1B WT from YN/YC-PTP1B WT. Src mutant Y529F was generated by PCR using the quick-change site-directed mutagenesis kit (Stratagene). The mRFP-rab11 plasmid was a kind gift of Dr. J. Lavoie, Université du Laval, Canada. The mRFP-sec24 was obtained by replacing the EYFP from pEYFP-C1-sec24 (provided by C. Alvarez, Universidad Nacional de Córdoba, Argentina) with mRFP excised from pmRFP-C1 (provided by Q. Wang and S. Green, University of Iowa, USA), using the Age I/Sal I restriction enzymes. Lck-mCherry was kindly provided by Steve Green (University of Iowa).

### Cell Culture, Transfection, and Live Cell Labeling

CHO-K1 cells and SYF cells were cultured in high glucose DMEM containing L-glutamine, supplemented with 10% fetal bovine serum, penicillin and streptomycin (Invitrogen, Carlsbad, CA). Transient transfections were performed in 24-well tissue culture plates using Lipofectamine 2000 (Invitrogen Corp, Carlsbad, CA, USA) and 0.5 µg of each construct per well. After 24 h, cells were resuspended and plated at a lower density on fibronectin (20 µg/ml)-coated coverslips. Labeling of live cells with vybrant DiO previous to the isolation of ventral membrane preparations was performed according to the manufacturer’s instructions (Invitrogen Corp, Carlsbad, CA, USA). DiO was analyzed using a Nikon B-2E/C filter cube.

### Antibodies and Other Labeling Reagents

The monoclonal antibody against PTP1B (used at 1/1000 dilution) was from BD Biosciences (Franklin Lakes, NJ). Polyclonal antibody against Src (used at 1/1000 dilution) was from Biosource (Camarillo, CA), polyclonal anti-Fyn (used at 1/500 dilution) was from Santa Cruz Biotechnology (Santa Cruz, CA), polyclonal anti-calnexin (used at 1/500 dilution), monoclonal anti-alpha tubulin (clone DM1A, used at 1/20.000), and Phalloidin-TRITC (used at 1/700) were from Sigma-Aldrich (St. Louis, MO). Secondary antibodies for Western blotting (used at 1/20.000 dilution) were from Jackson Immunoresearch (West Grove, PA) and Alexa Fluor488- and Fluor 568-conjugated secondary antibodies (used at 1/700 dilution) were from Invitrogen (Carlsbad, CA).

### Western Blots

Cells were lysed on ice with TBS (20 mM Tris-HCl, pH 7.4, 137 mM NaCl) containing 1% Triton X-100 and a protease inhibitor cocktail from Sigma-Aldrich (St. Louis, MO). Cell lysates were centrifuged at 13.600×g for 15 min at 4°C and ∼30 µg of the supernatants were fractionated by SDS-PAGE and transferred to polyvinyl difluoride membranes. Blots were probed with anti-PTP1B, anti-Src or anti-Fyn followed by peroxidase-conjugated secondary antibodies and revealed by enhanced chemiluminiscence.

### Immunofluorescence, Surface Reflectance Interference Contrast (SRIC) and Total Internal Reflection Fluorescence Microscopy (TIRFM)

Cells were seeded on fibronectin-coated (20 µg/ml) coverglasses (Marienfeld, Lauda-Königshofen, Germany) and processed for immunofluorescence 16–18 h later. All procedures were performed at room temperature and the dilution buffer used was PBS (137 mM NaCl, 2.7 mM KCl, 10 mM Na_2_HPO_4_, 1.8 mM KH_2_PO_4_, pH 7.4). Cells were sequentially fixed with 4% paraformaldehyde (20 min), permeabilized with 0.5% Triton X-100 (5 min) and blocked with 3% BSA (1 h). Incubations with the primary and secondary antibodies were carried out in a humid chamber for 1 h. Samples were mounted in Vectashield (Vector, Burlingame, CA) and observed through a 60x/1.4 NA objective in a Nikon TE2000-U microscope (Melville, NY) coupled to an Orca-AG cooled CCD camera (Hamamatsu Photonics, Hamamatsu, Japan). BiFC was analyzed with an excitation filter of 500/20 nm, an emission filter of 535/30 nm and a 86002v2bs dicroic mirror (Chroma Technology, Rockingham, VT). In cells expressing mRFP fusion proteins or immunolabeled, BiFC and red signals were discriminated using the following Nikon filter sets: for BiFC, excitation 480/30 nm, emission 535/40 nm, 505 (LP) dicroic mirror; for mRFP or Alexa Fluor 568 nm, excitation 540/25 nm, emission 620/60 nm, 565 (LP) dicroic mirror. To visualize cells by SRIC, a cube (Nikon) with a green excitation filter, a UV dicroic mirror and without barrier filter was set in place in the epi-filter rotating turret. The contrast of the SRIC image was optimized by partial closing the aperture and field diaphragms in front of the 100 W mercury lamp. Co-localization analysis shown in Figure 2 and Figure S3 was performed using the JACoP plugin of ImageJ [64]. Images for co-localization were background-subtracted using a rolling ball of 5 pixels. This processing cleaned the intracellular background without removing puncta that are relevant for the co-localization analysis. Correlation between the green/red pixels was visually inspected in a cytofluorogram and the spread of this distribution with respect to the fitted line was estimated by calculating the Pearson correlation (PC) coefficient. PC varies from -1 to 1, -1 corresponds to inverse correlation, 0 absence of correlation and 1 a full correlation. Quantification was estimated calculating M1 and M2 Manders coefficients [64]. *M*1 and *M*2 are built by summing intensities of co-localizing pixels from one channel and dividing it by its integrated density. A pixel from channel A is considered as co-localized if it has a non zero intensity counterpart in the channel B.

For TIRFM cells were adhered on fibronectin-coated 25 mm diameter coverglasses (Marienfeld, Lauda-Königshofen, Germany) and mounted on the stage of a fully motorized Nikon TE2000-E inverted microscope equipped for SRIC, epifluorescence, and TIRFM. In all cases cells were visualized through a 60×1.45 NA objective and imaged using an ORCA II ER CCD camera controlled by the Metamorph software (Molecular Devices, Downington, PA). For SRIC and wide-field observation cells were illuminated with a 100 W mercury lamp. For TIRFM cells were illuminated using a 488 nm argon laser. Evanescent wave penetration depth was calculated to be ∼210 nm by use of the ImageJ plugin “Calc TIRF” (written by Sebastian Rhode, Institute for Biophysics, SDT Group, University of Linz), using the following parameters: 488 nm as λ, 1.52 as n1, 1.33 as n2, and 62° as the incident light beam angle.

### Ventral Membrane Preparations

We basically followed the procedures described by Drees et al. [62] with a brief modification [63]. The method consists of hypotonic swelling of cells followed by brief sonication, which results in the removal of the dorsal (apical) cell surface, nucleus and intracellular membranes and organelles, leaving behind the ventral (basal) membrane attached to the substrate. Cells plated on fibronectin-coated coverslips placed in 35 mm tissue culture dishes were rinsed once in hypotonic buffer containing 1 mM ZnCl_2_ and incubated in the same buffer for 2 minutes at room temperature [63]. Cells were then transferred to an ice bead and incubated for additional 5 minutes. The culture dish was filled nearly to the brim with cold hypotonic buffer and placed on an adjustable platform. The dish was raised until the tip of the 1/8-in diameter microprobe of a Branson Sonifier 250 was 4–8 mm above the cells. Cells were sonicated with a brief (less than 1 second) pulse, duty cycle 20, and output 20%. Cells were rinsed in Ringer’s buffer before fixation with paraformaldehyde 4% in Ringer’s buffer for 20 minutes at room temperature.

### Time-lapse Imaging

Cells were seeded at ∼30% confluence on fibronectin-coated coverslips attached to the bottom of 35 mm dishes. Twenty four hours later dishes were placed on the stage of a Nikon TE2000-U inverted microscope enclosed within an incubator system (Solent Scientific Ltd, Fareham, UK) and the temperature was maintained at 37°C. Imaging medium was phenol red-free DMEM with high-glucose, supplemented with 4 mM L-glutamine and 25 mM Hepes buffer, 10% fetal bovine serum, and antibiotics. The medium also contained 0.5 U/ml oxyfluor (Oxyrase, Inc., Mansfield, OH) to prevent photobleaching and photodamage. Cells were imaged with a 60x/1.4 NA Plan Apo objective. The excitation light was attenuated using ND8 neutral density filters. Images were captured every 30 sec with an Orca-AG cooled CCD camera (Hamamatsu Photonics, Hamamatsu, Japan) using 2×2 binning. The exposure time was 2 sec. YFP was detected using filters (Chroma Technology Corp, Brattleboro, VT) placed in filter wheels (excitation 500/20, emission 535/30), using a 86007 bs dichroic mirror. Illumination was shuttered using a SmartShutter controlled by Lambda 10-B (Sutter Instrument, Novato, CA). Under our experimental conditions, we did not detect significant photobleaching. All peripherals were controlled with Metamorph 6.1 software (Molecular Devices, Downingtown, PA). Stacks of image sequences were built using the Metamorph or the ImageJ software (Wayne Rasband, NIH, Bethesda, MD). For display purposes images were processed for unsharp masking. Briefly, a blur image (sigma radius of 4) was subtracted from the original and the contrast and brightness were adjusted in the resulting image. Plot profiles of BiFC intensity were obtained from raw data with simple background subtraction.

## Supporting Information

Figure S1 **BiFC signal and Src expression levels.** CHO-K1 cells were co-transfected with the YC-PTP1BWT/Src-YN BiFC pair and then fixed and processed for immunofluorescence detection of Src using Alexa Fluor 568-conjugated secondary antibodies. (A) Src signal, (B) BiFC labeling. Cells 1 and 2 are transfected and display BiFC; cell 3 is not transfected and as result is BiFC negative. Background-subtracted images were used to draw line scans (yellow) over equivalent lamellar regions of cells 1, 2 and 3. Plot profiles corresponding to fluorescence intensity of line scans are shown at the bottom. Note that transfected cells 1 and 2 display roughly 2- and 4-fold higher levels of Src label compared to the non transfected cell 3. However, BiFC levels of cells 1 and 2 are similar in intensity. Scale bar, 30 µm.
(TIF)

Figure S2 **Distribution of the BiFC signal produced by Fyn constructs.** CHO-K1 cells were co-transfected with BiFC pairs and analyzed by fluorescence microscopy. Cells were also processed for immunofluorescence detection of PTP1B, using Alexa Fluor 568-conjugated secondary antibodies. BiFC distributions of (A) YC-PTP1BWT/Fyn-YN and (C) YC-PTP1BDA/Fyn-YN pairs. Note that in both cases the BiFC signal is punctate and spread throughout the cell. (B, D) PTP1B immuno-labeling. Dashed lines indicate the perimeter of cells. Scale bar, 20 µm.
(TIF)

Figure S3 **BiFC co-localization with Rab11, sec24, and dynamics in live cells.** (A–C) Cells expressing the YC-PTP1BDA/Src-YN BiFC pair (A, C) along with the marker of recycling endosomes mRFP-rab11 (B, C). The merge panel (C) was processed by unsharp masking and histogram stretching to enhance puncta visualization. The lack of co-localization is evident. Scale bar: 25 µm. (E-G) Analysis of BiFC in live cells. CHO-K1 cells were co-transfected with YC-PTP1BWT/Src-YN (E) or co-transfected with YC-PTP1BDA/Src-YN and mRFP-sec24 (F, G). Images of live cells were acquired 24 h post-transfection using an inverted Nikon TE2000 microscopy system fitted for time-lapse analysis (see Materials and Methods). As observed with fixed cells (Fig. 2), the BiFC signal produced by PTP1BWT (E) follows a network pattern with overlapping puncta while that produced by the substrate trap mutant DA (F) shows mainly puncta. (G) The merge image of BiFC and mRFP-sec24 was processed as in (C) and reveals a lack of co-localization between the two signals. (D and H) Cytofluorograms representing the low correlation between BiFC images and either mRFP-rab11 (C) or mRFP-sec24 (G) are shown. Arbitrary units (a.u) represent grey level values from 12-bit images. Pearson’s correlation coefficient approaches to zero in both cases revealing a lack of co-localization. This fact is also indicated by the low percentages of Manders’ coefficients M1 and M2. (I, J) Time-lapse analysis of CHO-K1 cells expressing YC-PTP1BDA/Src-YN. Cells were imaged every 30 sec during 10 min. Numbers indicate min. (I) Two frames showing how the BiFC label of one punctum is redistributed between two puncta (compare intensity of “b” at 0′ with that of “b′” at 3′). Intensity line scans drawn between arrowheads are shown in the plot at the right. Note that during the same period the intensity of the BiFC puncta marked with “a” and “a′” did not change. (J) Selected frames of a time lapse series showing the dynamics of tubules connecting spatially close puncta (arrowheads). The square box is 6 µm per side. Images in panels I and J were processed by unsharp masking and histogram stretching to enhance puncta visualization. The associated plots were generated using raw data from background-subtracted images.
(TIF)

Figure S4 **Analysis by TIRFM and time lapse of GFP-PTP1BDA.** CHO-K1 cells were transfected with GFP-PTP1BDA and then fixed and analyzed by wide field (A, wf) and TIRFM (B-B″). (B′ and B″) are 500% magnifications of yellow boxes shown in (B). Images were processed by unsharp masking and histogram stretching to enhance puncta visualization. Note the comet-like fluorescence of the fluorescence (yellow arrowheads in B′ and B″). (C) Selected frames of time-lapse analysis of living cells expressing GFP-PTP1BDA. Images of live cells were taken every 2 min. In three frames it is shown how the fluorescence intensity of GFP-PTP1BDA puncta is redistributed from two puncta (“a” at 0′) to one (“b” at 4′) and then split into two puncta again (“c” at 8′). Images were processed as described in Figure 4. Intensity line scans drawn between arrowheads are shown in the plot at the right. Scale bar, 20 µm.
(TIF)

Figure S5 **Ventral membrane preparations.** (A, B) Whole CHO-K1 cells were fixed and triple stained with anti-calnexin/Alexa Fluor 488-conjugated secondary antibodies, DAPI, and either Phalloidin-TRITC (A) or anti-alpha tubulin/Alexa Fluor 568-conjugated secondary antibodies (B). F-actin, microtubules, ER and nuclei had the typical staining. (C-H) These panels show ventral membrane preparations. (C, D) Ventral membranes were fixed and triple stained with anti-calnexin/Alexa Fluor488-conjugated secondary antibodies, DAPI, and either Phalloidin-TRITC (C) or anti-alpha tubulin/Alexa Fluor 568-conjugated secondary antibodies (D). Note that nuclei and most of the F-actin, microtubules and ER were removed. (E) Cells were labeled with the lypophilic dye vybrant DiO before preparation of ventral membranes and fixation. DiO fluorescence was observed under the green channel. (F) SRIC image corresponding to the same cell as in (E). Note the presence of the ventral membrane by the DiO fluorescence and the dark streaks revealing the adhesions to the substrate under the SRIC optics. (G, H) Cells were transfected with Src-YN and YC-PTP1BDA before preparation of ventral membranes and fixation. Src-YN (G) and YC-PTP1BDA (H) were detected by specific antibodies. Note the labeling in puncta for both proteins. The perimeter of cells is indicated by yellow dashed lines. Scale bar: 30 µm.
(TIF)

Figure S6 **Distribution of SrcT-YN in ventral membranes.** (A) Diagram showing the N-terminal deletion in SrcT-YN. The Glycine substrate of myristoylation is indicated by an arrowhead. Lysines of the polybasic motif are underlined. (B-E) CHO-K1 cells were co-transfected with SrcT-YN and Lck-mCherry before preparation of ventral membranes and fixation. Nuclei were labeled with DAPI. Note the absence of SrcT-YN puncta (B) and nucleus (C) but the retention of Lck-mCherry (D) in the ventral membranes. (E) SRIC image. Scale bar: 30 µm.
(TIF)

Movie S1 **Dynamics of BiFC puncta and tubules.** This movie relates to Figure S3 of the manuscript. CHO-K1 cells were co-transfected with the YC-PTP1BDA/Src-YN pair. A day later cells were imaged every 30 sec for a total time of 10 min. White arrows point tubular links between puncta. Movie runs at 5 fps; movie frame, 312×332 (width/height) pixels.
(MOV)
