# Supplementary figures and images for: ER-Bound Protein Tyrosine Phosphatase PTP1B Interacts with Src at the Plasma Membrane/Substrate Interface
Source: PLoS One. 2012 Jun 11;7(6):e38948. doi: 10.1371/journal.pone.0038948 (PMC3372476; doi:10.1371/journal.pone.0038948)

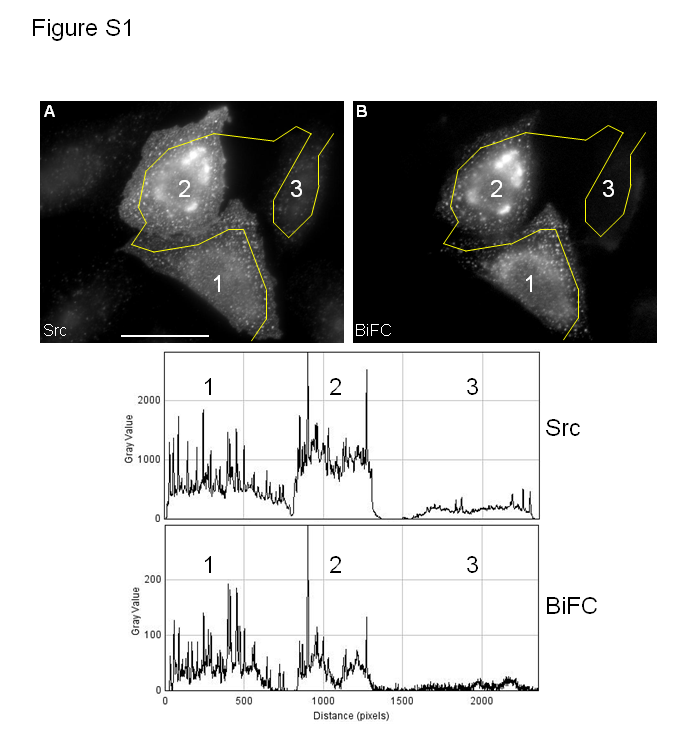

Supplement: Figure S1 — BiFC signal and Src expression levels. CHO-K1 cells were co-transfected with the YC-PTP1BWT/Src-YN BiFC pair and then fixed and processed for immunofluorescence detection of Src using Alexa Fluor 568-conjugated secondary antibodies. (A) Src signal, (B) BiFC labeling. Cells 1 and 2 are transfected and display BiFC; cell 3 is not transfected and as result is BiFC negative. Background-subtracted images were used to draw line scans (yellow) over equivalent lamellar regions of cells 1, 2 and 3. Plot profiles corresponding to fluorescence intensity of line scans are shown at the bottom. Note that transfected cells 1 and 2 display roughly 2- and 4-fold higher levels of Src label compared to the non transfected cell 3. However, BiFC levels of cells 1 and 2 are similar in intensity. Scale bar, 30 µm. (TIF) [file pone.0038948.s001.tif]

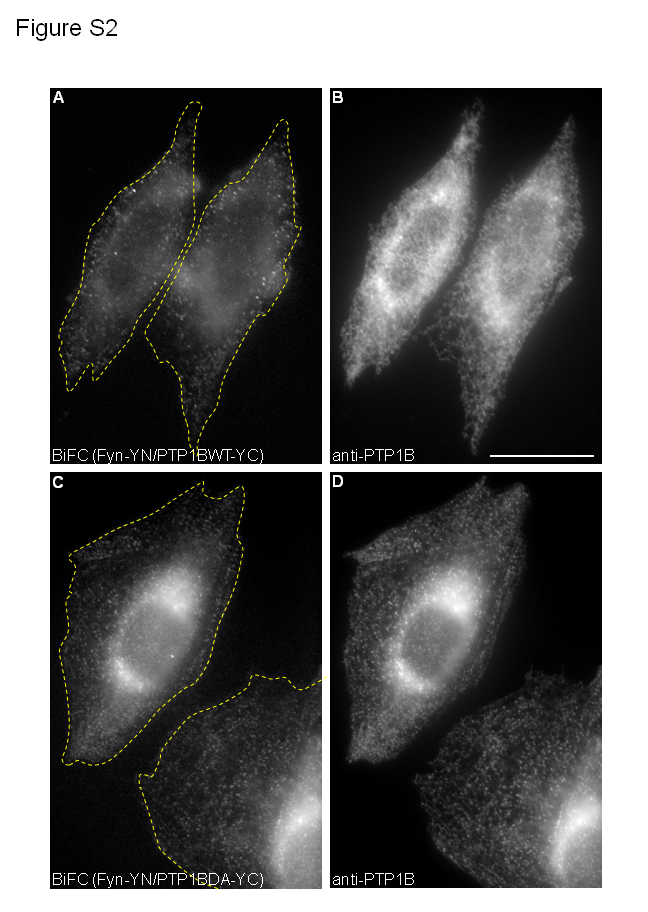

Supplement: Figure S2 — Distribution of the BiFC signal produced by Fyn constructs. CHO-K1 cells were co-transfected with BiFC pairs and analyzed by fluorescence microscopy. Cells were also processed for immunofluorescence detection of PTP1B, using Alexa Fluor 568-conjugated secondary antibodies. BiFC distributions of (A) YC-PTP1BWT/Fyn-YN and (C) YC-PTP1BDA/Fyn-YN pairs. Note that in both cases the BiFC signal is punctate and spread throughout the cell. (B, D) PTP1B immuno-labeling. Dashed lines indicate the perimeter of cells. Scale bar, 20 µm. (TIF) [file pone.0038948.s002.tif]

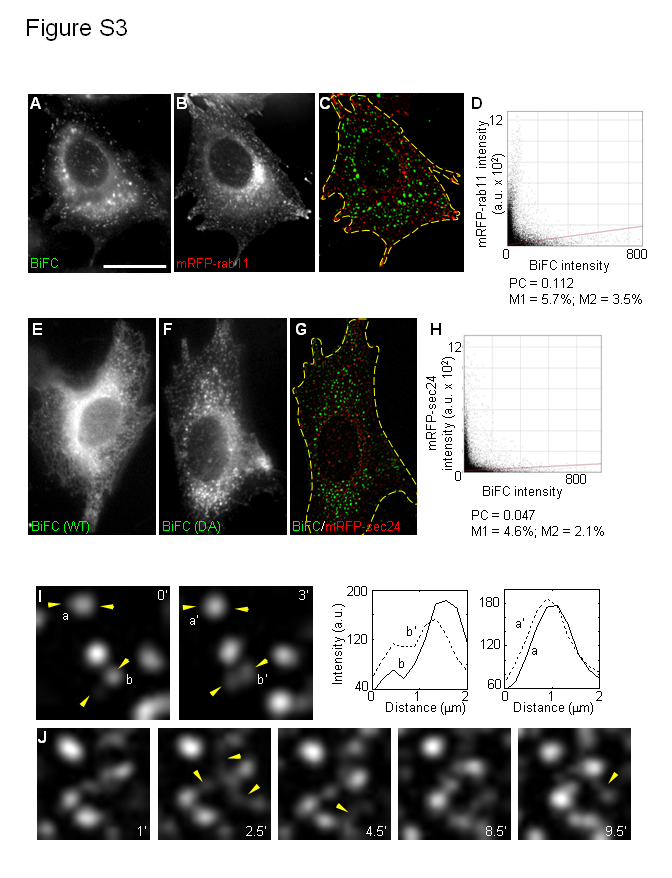

Supplement: Figure S3 — BiFC co-localization with Rab11, sec24, and dynamics in live cells. (A–C) Cells expressing the YC-PTP1BDA/Src-YN BiFC pair (A, C) along with the marker of recycling endosomes mRFP-rab11 (B, C). The merge panel (C) was processed by unsharp masking and histogram stretching to enhance puncta visualization. The lack of co-localization is evident. Scale bar: 25 µm. (E-G) Analysis of BiFC in live cells. CHO-K1 cells were co-transfected with YC-PTP1BWT/Src-YN (E) or co-transfected with YC-PTP1BDA/Src-YN and mRFP-sec24 (F, G). Images of live cells were acquired 24 h post-transfection using an inverted Nikon TE2000 microscopy system fitted for time-lapse analysis (see Materials and Methods). As observed with fixed cells (Fig. 2), the BiFC signal produced by PTP1BWT (E) follows a network pattern with overlapping puncta while that produced by the substrate trap mutant DA (F) shows mainly puncta. (G) The merge image of BiFC and mRFP-sec24 was processed as in (C) and reveals a lack of co-localization between the two signals. (D and H) Cytofluorograms representing the low correlation between BiFC images and either mRFP-rab11 (C) or mRFP-sec24 (G) are shown. Arbitrary units (a.u) represent grey level values from 12-bit images. Pearson’s correlation coefficient approaches to zero in both cases revealing a lack of co-localization. This fact is also indicated by the low percentages of Manders’ coefficients M1 and M2. (I, J) Time-lapse analysis of CHO-K1 cells expressing YC-PTP1BDA/Src-YN. Cells were imaged every 30 sec during 10 min. Numbers indicate min. (I) Two frames showing how the BiFC label of one punctum is redistributed between two puncta (compare intensity of “b” at 0′ with that of “b′” at 3′). Intensity line scans drawn between arrowheads are shown in the plot at the right. Note that during the same period the intensity of the BiFC puncta marked with “a” and “a′” did not change. (J) Selected frames of a time lapse series showing the dynamics of tubules connecting spatially [file pone.0038948.s003.tif]

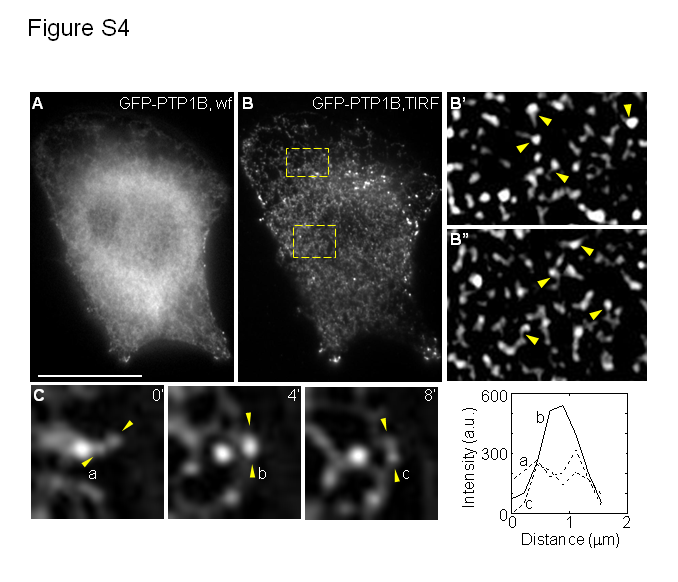

Supplement: Figure S4 — Analysis by TIRFM and time lapse of GFP-PTP1BDA. CHO-K1 cells were transfected with GFP-PTP1BDA and then fixed and analyzed by wide field (A, wf) and TIRFM (B-B″). (B′ and B″) are 500% magnifications of yellow boxes shown in (B). Images were processed by unsharp masking and histogram stretching to enhance puncta visualization. Note the comet-like fluorescence of the fluorescence (yellow arrowheads in B′ and B″). (C) Selected frames of time-lapse analysis of living cells expressing GFP-PTP1BDA. Images of live cells were taken every 2 min. In three frames it is shown how the fluorescence intensity of GFP-PTP1BDA puncta is redistributed from two puncta (“a” at 0′) to one (“b” at 4′) and then split into two puncta again (“c” at 8′). Images were processed as described in Figure 4. Intensity line scans drawn between arrowheads are shown in the plot at the right. Scale bar, 20 µm. (TIF) [file pone.0038948.s004.tif]

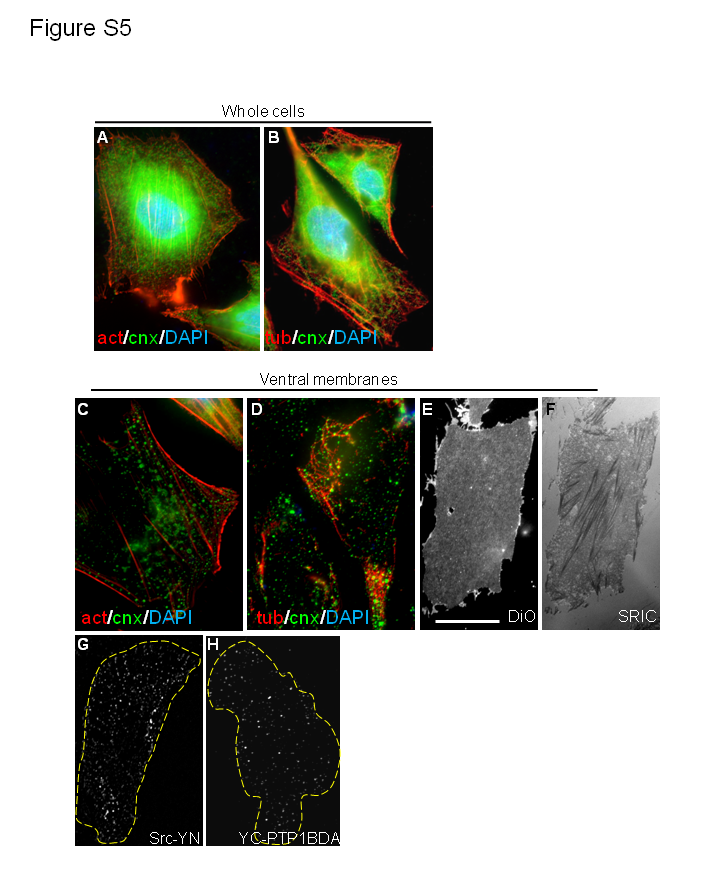

Supplement: Figure S5 — Ventral membrane preparations. (A, B) Whole CHO-K1 cells were fixed and triple stained with anti-calnexin/Alexa Fluor 488-conjugated secondary antibodies, DAPI, and either Phalloidin-TRITC (A) or anti-alpha tubulin/Alexa Fluor 568-conjugated secondary antibodies (B). F-actin, microtubules, ER and nuclei had the typical staining. (C-H) These panels show ventral membrane preparations. (C, D) Ventral membranes were fixed and triple stained with anti-calnexin/Alexa Fluor488-conjugated secondary antibodies, DAPI, and either Phalloidin-TRITC (C) or anti-alpha tubulin/Alexa Fluor 568-conjugated secondary antibodies (D). Note that nuclei and most of the F-actin, microtubules and ER were removed. (E) Cells were labeled with the lypophilic dye vybrant DiO before preparation of ventral membranes and fixation. DiO fluorescence was observed under the green channel. (F) SRIC image corresponding to the same cell as in (E). Note the presence of the ventral membrane by the DiO fluorescence and the dark streaks revealing the adhesions to the substrate under the SRIC optics. (G, H) Cells were transfected with Src-YN and YC-PTP1BDA before preparation of ventral membranes and fixation. Src-YN (G) and YC-PTP1BDA (H) were detected by specific antibodies. Note the labeling in puncta for both proteins. The perimeter of cells is indicated by yellow dashed lines. Scale bar: 30 µm. (TIF) [file pone.0038948.s005.tif]

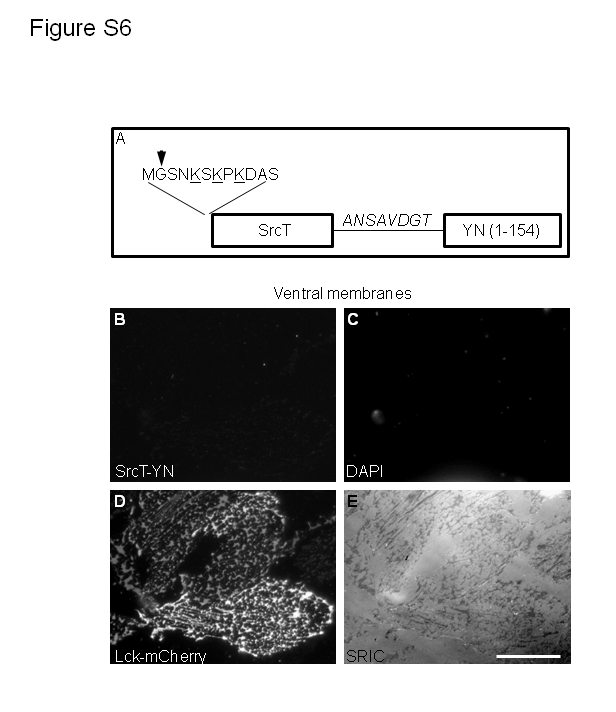

Supplement: Figure S6 — Distribution of SrcT-YN in ventral membranes. (A) Diagram showing the N-terminal deletion in SrcT-YN. The Glycine substrate of myristoylation is indicated by an arrowhead. Lysines of the polybasic motif are underlined. (B-E) CHO-K1 cells were co-transfected with SrcT-YN and Lck-mCherry before preparation of ventral membranes and fixation. Nuclei were labeled with DAPI. Note the absence of SrcT-YN puncta (B) and nucleus (C) but the retention of Lck-mCherry (D) in the ventral membranes. (E) SRIC image. Scale bar: 30 µm. (TIF) [file pone.0038948.s006.tif]
